# 4151 Understanding barriers and solutions towards access to mental health among rural adolescents

**DOI:** 10.1017/cts.2020.443

**Published:** 2020-07-29

**Authors:** Brandy Davis, Kimberly B. Garza, Salisa Westrick, Edward Chou, Cherry Jackson

**Affiliations:** 1 University of Alabama at Birmingham; 2 Auburn University

## Abstract

OBJECTIVES/GOALS: There are two objectives: 1) To identify healthcare providers’ (HCP) barriers and potential solutions towards rural adolescents’ access to mental healthcare. Healthcare providers include pharmacists, physicians, and mental healthcare providers (MHPs). 2) To identify rural high schoolers’ barriers and potential solutions towards access to mental healthcare. METHODS/STUDY POPULATION: Fifteen HCPs will be recruited via email listserv and the snowball method. Perceived barriers of rural adolescents, personal barriers, current practices to address mental health in adolescents, and preferred solutions will be discussed. Twenty student and parent dyads will be recruited using fliers in school systems and will be interviewed individually outside of class time on school grounds or over the phone. Barriers to care and preferred solutions will be discussed. All interviews will be semi-structured, recorded, conducted in person or over the phone, and last for 30 minutes to an hour. Compensation will be $25 for students and parents each, $50 for pharmacists and mental health providers and $100 for physicians. Thematic qualitative data analysis will be performed using Atlas.ti software. RESULTS/ANTICIPATED RESULTS: Data collection is ongoing. Anticipated results for barriers include absence of mental healthcare providers in rural areas, inability to access mental healthcare providers further away, stigma towards mental healthcare, and lack of knowledge of mental health conditions and treatment. Anticipated results for potential solutions may include promoting mobile applications to assist with telehealth and self-care. Other solutions may be collaboration among rural healthcare providers for adolescents with mental health conditions. Preferred solutions may also include pharmacists disseminating knowledge to rural adolescents and their parents or referrals to mental healthcare providers. DISCUSSION/SIGNIFICANCE OF IMPACT: This project will identify barriers and solutions to access to mental healthcare among rural adolescents. These solutions can then be applied towards the creation of programs that address salient issues within rural communities with a greater chance of uptake and use so that rates of depression and suicide will decrease. CONFLICT OF INTEREST DESCRIPTION: Funding through UAB TL1 award.

